# Prevalence of mental disorders among Australian females: Comparison according to motherhood status using Australian birth cohort data

**DOI:** 10.1007/s00737-024-01444-2

**Published:** 2024-02-20

**Authors:** Diksha Sapkota, James Ogilvie, Susan Dennison, Carleen Thompson, Troy Allard

**Affiliations:** 1 Griffith Criminology Institute, Griffith University, 176 Messines Ridge Road, Mount Gravatt, Brisbane, QLD 4122 Australia; 2 School of Criminology and Criminal Justice, Griffith University, Brisbane, Australia

**Keywords:** Longitudinal, Maternal, Mental illness, Prevalence

## Abstract

**Purpose:**

Studies examining mental disorders among women have primarily focused on either depression, anxiety, or substance use disorders and not included the broader spectrum of mental disorders. Mixed evidence exists on the prevalence rates of mental disorders among mothers. This study compares the prevalence of different mental disorders and mental comorbidities between mothers and non-mothers and assesses correlates of mental disorders among mothers.

**Methods:**

A population-based birth cohort design was adopted, consisting of 40,416 females born in Queensland, Australia, in 1983/84. Linked administrative data from hospital admissions were used to identify mental disorders. Cumulative incidence curves of different mental disorders were created separately for mothers and non-mothers.

**Results:**

Mental disorder prevalence among females by age 29–31 years was 7.8% (11.0% for mothers and 5.2% for non-mothers). Mothers were overrepresented in almost all categories of mental disorders, with overrepresentation becoming more pronounced with age. Mothers with a mental disorder were more likely to be unmarried, Indigenous, young at birth of first child, have greater disadvantage, and have a single child, compared to mothers without a mental disorder. Nearly half of the mothers (46.9%) had received a mental disorder diagnosis before having their first child.

**Conclusions:**

Mothers, particularly unmarried, Indigenous, having greater disadvantage, and younger at birth of first child, represent a unique group with high vulnerability to mental disorders, that begins in childhood and is amplified with age. Presence of significant mental disorder comorbidities among females highlights the critical importance of a comprehensive, integrated approach to prevent and address multiple comorbidities.

**Supplementary information:**

The online version contains supplementary material available at 10.1007/s00737-024-01444-2.

## Introduction

Extensive research has documented the significant burden of mental disorders among females (Otten et al. 2021; GBD 2019 Mental Disorders Collaborators 2022), but available evidence is often limited to cross-sectional studies exploring prevalence estimates of either one or a few common mental disorders. Although most women are likely to become mothers during their lifetime, limited information exists about the prevalence of mental disorders among mothers. Longitudinal, population-based data can provide reliable estimates of the prevalence of the full range of mental disorders and factors associated with these disorders. This paper reports the prevalence of mental disorders among females included in 1983/84 Queensland, Australia, birth cohorts and compares the prevalence rates between mothers and women without children (hereafter referred to as *non-mothers*). It further explores socio-demographic and maternal factors associated with mental disorders among mothers.

Motherhood is recognised as a significant event in a woman’s life and there are inconsistent findings regarding mental health of mothers and non-mothers. For some mothers, having children leads to feelings of connectedness, self-fulfilment, personal growth, and satisfaction; thus, improving their psychological wellbeing (Holton et al. 2010; Graham 2015; Kuipers et al. 2021). While for some, the transition to motherhood has been linked to increased stress, loss of freedom, and poor emotional health because of several physiological changes during pregnancy and childbirth, as well as psychosocial challenges and lifestyle changes related to childrearing (Munk-Olsen et al. 2006; Gonçalves et al. 2016; Pearson et al. 2019). Hence, there is lack of consensus about prevalence rates for mothers and non-mothers, particularly in relation to mental disorders other than depression, anxiety, and substance use disorders (SUDs), which have been the dominant focus of research to date (Holden et al. 2019; Otten et al. 2021).

Available evidence on maternal mental health have mostly focused on depression, anxiety, or postpartum traumatic stress disorder among pregnant and postpartum women (within 12 months of birth), utilised cross-sectional surveys that were either unrepresentative, methodologically weaker (e.g., use of mental health symptoms screeners, lack of comparison groups, and small sample size) (Woolhouse et al. 2014; Howard and Khalifeh 2020). Such methodological variation and differences in socio-cultural background of mothers from different contexts have resulted in wide discrepancies of prevalence rates across studies (Sapkota et al., accepted for publication; Bezerra et al. 2021). In addition, there is limited, yet mixed, evidence regarding whether mental disorders are more likely to occur before or after childbirth. For example, in a study by Patton et al. (2015), depression was documented to precede motherhood; however, another population-based study documented an increased risk of mental health contacts in the first three months following the childbirth (Munk-Olsen et al. 2006). There are also debates around factors contributing to mental disorders among mothers. A study from Australia found that maternal depression was more common among mothers with one child (Woolhouse et al. 2014), while another study reported poor mental health among older mothers with multiple children (Grundy et al. 2019). The relationship between number of children and mental disorders over time is inconsistent and seems to be affected by other factors, such as marital status, socio-economic status, and lifestyle factors (Grundy et al. 2019).

Existing literature documents a strong pattern of comorbidity across mental disorders (Barr et al. 2022) and an increased risk of developing subsequent disorders in later life among women with existing mental disorders (Plana-Ripoll et al. 2019). For example, among women with a mood disorder before age 20, 50% were likely to develop anxiety disorders within the next 15 years, with stronger associations in the first 6 months after receiving a diagnosis of mood disorders (Plana-Ripoll et al. 2019). Past studies on comorbidity are limited to specific geographic regions or subsets of mental disorders and/or have relied on self-reports (Ottosen et al. 2016; Plana-Ripoll et al. 2019; Konrad et al. 2022). To our knowledge, no published studies have documented the differences in mental disorder comorbidities between mothers and non-mothers using population-based representative data.

Overall, there is an absence of accurate and generalisable estimates of rates of diagnosed mental disorders among females and it is unclear how mothers differ from non-mothers in terms of mental disorders. In this study, we focused on women born in Queensland in the year 1983/84 and used hospital admissions data to answer the following research questions: 1) Do mothers have higher or lower prevalences rates of mental disorders compared to non-mothers? 2) Is there a difference in the sociodemographic and mental health profiles, including mental comorbidities, between mothers and non-mothers? 3) Does a mental disorder diagnosis occur before or after the first childbirth? and 4) What are the sociodemographic and maternal correlates of mental disorders among mothers?

## Methodology

### Data sources and participants

Data were derived from the Queensland Cross-sector Research Collaboration (QCRC) repository, which includes population-based de-identified linked administrative data from multiple government agencies for all individuals born in 1983 and 1984 in the state of Queensland, Australia (see Stewart et al. 2015, 2021 for further information, with details on the data linkage process provided in the latter paper). This study included data from the Queensland Registry of Births, Deaths, and Marriages (RDBM) and the Queensland Hospital Admitted Patient Data Collection (QHAPDC). The current study adopted the STrengthening the Reporting of OBservational studies in Epidemiology (STROBE) guidelines (von Elm et al. 2014).

The current study included females born in Queensland in 1983 and 1984, consisting of 40,416 females followed up to age 29/30/31 years. There were 299 deaths recorded for the cohort (0.7%), with 25.1% of these deaths occurring during the first year after birth. The earliest age of diagnosis of any mental disorder was 10.8 years, so any deaths occurring before 10 years were excluded, resulting in a final sample of 40,294 females (45.1% mothers, out of which 9.7% were Indigenous).

### Outcomes and measures

Information on study variables and the data sources is summarised in Table 1.

**Table 1:** Study variables: Definitions and data sources

Study variables	Definition and data sources
Mental disorder diagnosis-related variables	
Mental disorder	Mental disorder diagnoses were extracted from the QHAPDC, where they are classified according to the International Statistical Classification of Diseases and Related Health Problems – tenth edition Australian modification (ICD-10AM; World Health Organization 2004). QHAPDC records were available from 1 July 1995, as a result data were left-censored until age 11/12 years for the 1983 cohort and 10/11 years for the 1984 cohort. All diagnoses across all admission episodes were extracted for individuals up to June 2014, resulting in mental disorder diagnoses to age 30/31 for the 1983 cohort and 29/30 for the 1984 cohort. Therefore, mental disorder diagnoses were captured for a period of approximately 20 years duration Females were coded as having a mental disorder if they had experienced a hospital admission and received a diagnostic code for *mental and behavioural disorders* (ICD-10 codes F00 to F99), *suicidal ideation* (code R45.8) or *self-harm* (codes X60 to X84]) as either a primary or additional diagnosis. Mental disorders were classified into seven broad diagnostic groups (i.e., mood; anxiety; psychotic; personality; substance use; other adult-onset; and child-onset disorders), and then further subdivided into more detailed subcategories/subdivisions (see Supplementary Table S1 for details of broad and subcategories of mental disorders along with their corresponding ICD-10 codes). All categories included mutually exclusive sets of ICD-10 diagnoses and were coded as present/not present for each female. Individuals could appear in more than one diagnostic category/subdivision if they received diagnoses across different diagnostic categories/ subdivisions
Comorbidity and dual diagnosis	Comorbidity was defined as the occurrence of at least two mental disorders from different subdivisions (except SUDs), while females with a positive history of SUDs and another mental disorder were coded as having a dual diagnosis
Age at first hospitalisation	Computed using the individual’s birth date and their date of first mental health related admission from the QHAPDC
Sociodemographic variables	
Mother status	Any female registered as having as least one child in the RBDM records was coded as a “mother” (1) and those without any registered children as a “non-mother” (0)
Marital status	Ever married (1)/Never married (0) in the RBDM records
Indigenous status	Individual who ever identified as Indigenous Australian (Aboriginal and/or Torres Strait Islander) in any of the QCRC databases was considered Indigenous Australian (1)
Remoteness of usual place of residence at first hospital admission	Computed using usual place of residence at first mental health-related hospital admission and coded according to the Australian Standard Geographical Classification – Remoteness Area classification (Australian Bureau of Statistics 2021-2026) as a binary variable (regional and remote areas [1]; major cities [2])
Index of relative socio-economic advantage and disadvantage (IRSAD)	Computed using individuals’ usual place of residence at first mental-health related admission, derived from the Socio-Economic Indexes for Areas (SEIFA; only available for those with at least one mental disorder). Scores ranged from 1 to 10, with lower scores reflecting greater disadvantage and lack of advantage, while higher scores reflect a relative lack of disadvantage and greater advantage. For analysis among mothers, IRSAD was documented using the mother’s postcode at first childbirth (Australian Bureau of Statistics 2023) and categorised as Disadvantaged (1 to 5 score) [1] and advantaged (6 to 10 score) [2]. This information was available only for women with biological children
Maternal factors	
Age at first childbirth	Computed using the individual’s birth date and their first child’s birth date from the RBDM records. Women who delivered before 20 years of age were coded as having a history of teenage pregnancy (1)
Number of children	A categorical variable was created for number of biological children (1 child and ≥ 2 children) using the RBDM records

### Statistical analyses

Analyses were conducted using R version 3.2–3 (R Development Core Team 2019) and SPSS v 29.0 (IBM Corp. 2022).

#### Lifetime prevalence of mental disorders

Prevalence rates were calculated as the proportion of the cohort with any inpatient mental disorder diagnosis by age 29–31 years. Crude prevalence rates by diagnostic divisions and subdivisions are presented, along with chi-square tests (*χ*^2^) to explore differences across motherhood status. Sociodemographic and mental health profiles of females with a mental disorder were compared by motherhood status using a series of *χ*^2^ and Mann–Whitney U Test and a binary logistic regression. There were 106 females (0.3%) diagnosed with puerperium related disorders (F53 codes) who were excluded from analyses that compared mothers and non-mothers.

#### Cumulative incidence of mental disorders

Cumulative incidence was defined as the cumulative proportion of the cohort receiving their first mental health diagnosis, stratified by motherhood status, up to age 29–31. Cumulative incidence of first diagnosis by age was modelled using the survival package for R (Therneau and Lumley 2015). For deceased cases, age at death was taken as the point of censoring for the cumulative incidence analysis. 

#### Comorbidity of mental disorders

Tetrachoric correlations were used to explore the extent of comorbidity across broad diagnostic categories of mental disorders and separate analyses were conducted for mothers and non-mothers.

Sociodemographic and maternal factors of mothers with and without mental disorders were compared using *χ*^2^, Mann–Whitney U test, and a binary logistic regression.

## Results

### Lifetime prevalence of mental disorders by motherhood status

The population included 40,294 females (5.5% Indigenous, 38.4% ever married), of which 7.8% (*n* = 3,133) had at least one mental disorder. Lifetime prevalence estimates of any mental disorder, as well broad and subdivisional categories of hospital-diagnosed mental disorders diagnoses, varied significantly between mothers and non-mothers (Table 2). Compared to non-mothers, mothers had higher rates of any mental disorder (11.0% vs 5.2%) and all broad categories of mental disorders, except for child-onset disorders. Mothers were also overrepresented in most subdivisions of mental disorders, such as depression (3.4% vs. 1.7%), self-harm (2.9% vs 1.8%), and adjustment disorders (1.5% vs 0.8%). The most prevalent disorder was SUDs (5.0%) among mothers while it was anxiety among non-mothers (2.3%). We also compared the socio-demographic and mental health profiles of mothers and non-mothers with at least one mental disorder (see Supplementary Table S2). Compared to non-mothers, mothers with a mental disorder were more likely to be Indigenous, ever married, and residing on locations with higher levels of disadvantage. There were no significant differences between mothers and non-mothers in terms of common mental disorders except for psychotic, child-onset, and adult-onset disorders, with mothers having reduced odds of being diagnosed with these disorders that non-mothers.

**Table 2:** Prevalence rates of hospital-diagnosed mental disorders among females born in 1983/84 by their motherhood status (*n* = 40,294)

Hospital-diagnosed mental disorders	Total sample [n (%)]	% out of those with at least one mental disorder	All mothers [n (%)]	All non-mothers [n (%)]	*χ*^2^- value ^*a*^	*ϕ*_c_
	*n* = 40,294	*n* = 3,133	*n* = 18,188	*n* = 22,106		
Indigenous ^b^	2,197 (5.5)	18.2	1,762 (9.7)	435 (2.0)	1153.56***	0.17
Ever married ^c^	15,469 (38.4)	30.1	10,949 (60.2)	4,520 (20.4)	6666.59***	0.41
Any mental disorder	3,133 (7.8)	-	1,994 (11.0)	1,139 (5.2)	469.85***	0.11
Mood (Affective) disorders	1,087 (2.7)	34.7	683 (3.8)	404 (1.8)	141.26***	0.06
Depression	989 (2.5)	31.6	626 (3.4)	363 (1.6)	134.96***	0.06
Bipolar	157 (0.4)	5.0	86 (0.5)	71 (0.3)	5.91*	0.01
Other affective disorders	38 (0.1)	1.2	26 (0.1)	12 (0.1)	8.33**	0.01
Anxiety disorders	1,360 (3.4)	43.4	853 (4.7)	507 (2.3)	175.71***	0.07
Phobic anxiety disorders	72 (0.2)	2.3	42 (0.2)	30 (0.1)	5.07*	0.01
Reaction to severe stress	488 (1.2)	15.6	276 (1.5)	212 (1.0)	26.01***	0.03
Adjustment disorders	432 (1.1)	13.8	267 (1.5)	165 (0.8)	48.99***	0.04
Other anxiety disorders	702 (1.7)	22.4	462 (2.5)	240 (1.1)	123.31***	0.06
OCDs	57 (0.1)	1.8	30 (0.2)	27 (0.1)	1.29	0.01
Psychotic disorders	377 (0.9)	12.0	192 (1.1)	185 (0.8)	5.15*	0.01
Psychotic affective disorders	105 (0.3)	3.4	54 (0.3)	51 (0.2)	1.68	0.01
Psychotic disorders related to substance use	147 (0.4)	4.7	80 (0.4)	67 (0.3)	5.14*	0.01
Schizophrenia and delusional disorders	255 (0.6)	8.1	129 (0.7)	126 (0.6)	3.08	0.01
Personality disorders	388 (1.0)	12.4	213 (1.2)	175 (0.8)	15.07***	0.02
Substance use disorders	1,378 (3.4)	44.0	909 (5.0)	469 (2.1)	249.92***	0.08
Alcohol use disorder	907 (2.3)	29.0	562 (3.1)	345 (1.6)	106.06***	0.05
Other substance use disorder	773 (1.9)	24.7	540 (3.0)	233 (1.1)	194.47***	0.07
Other adult-onset disorders	1,090 (2.7)	34.8	625 (3.4)	465 (2.1)	67.35***	0.04
Eating disorders	130 (0.3)	4.2	63 (0.3)	67 (0.3)	0.58	< 0.01
Organic disorders	83 (0.2)	2.7	42 (0.2)	41 (0.2)	1.00	< 0.01
Self-harm	926 (2.3)	30.0	533 (2.9)	393 (1.8)	59.05***	0.04
Other adult disorders	60 (0.2)	1.9	38 (0.2)	22 (0.1)	8.03**	0.01
Child onset disorders	236 (0.6)	7.5	117 (0.6)	119 (0.5)	1.89	0.01
Intellectual impairment	94 (0.2)	3.0	32 (0.2)	62 (0.3)	4.68*	0.01
Developmental disorders	59 (0.2)	1.9	20 (0.1)	39 (0.2)	3.01	0.01
Behavioural and emotional disorders	129 (0.3)	4.1	79 (0.4)	50 (0.2)	13.55***	0.02

^a^Categorical variables were compared using Pearson *X*^2^ test

^b^Comparison group is non-Indigenous

^c^Comparison group is never married

*ϕ*_c_ Cramer’s V co-efficient

*p < 0.05, ** *p* < 0.01, *** *p* < 0.001

### Cumulative incidence of mental disorders

**Fig. 1:**
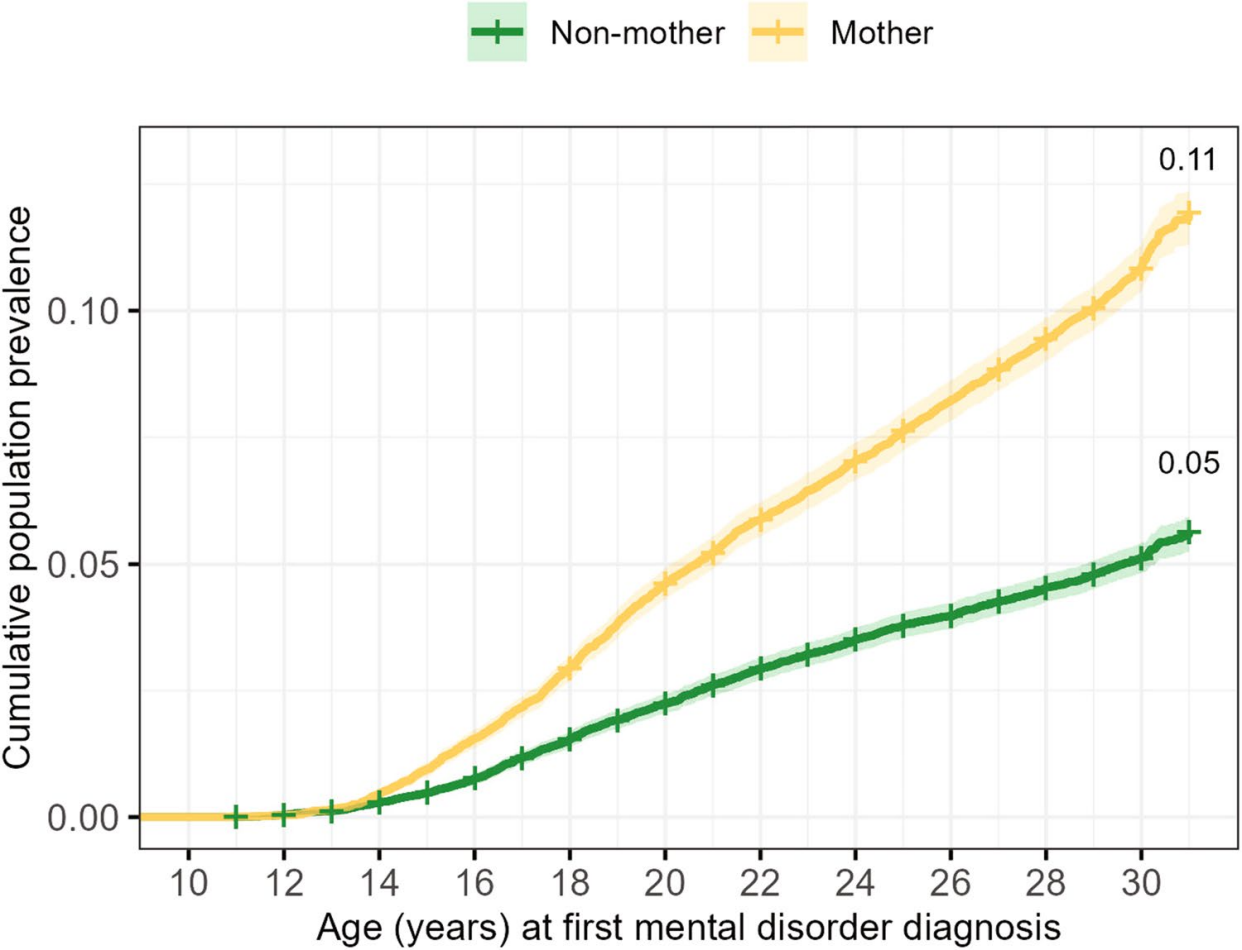
Cumulative prevalence of any mental disorders up to age 29–31 years by motherhood status

**Fig. 2:**
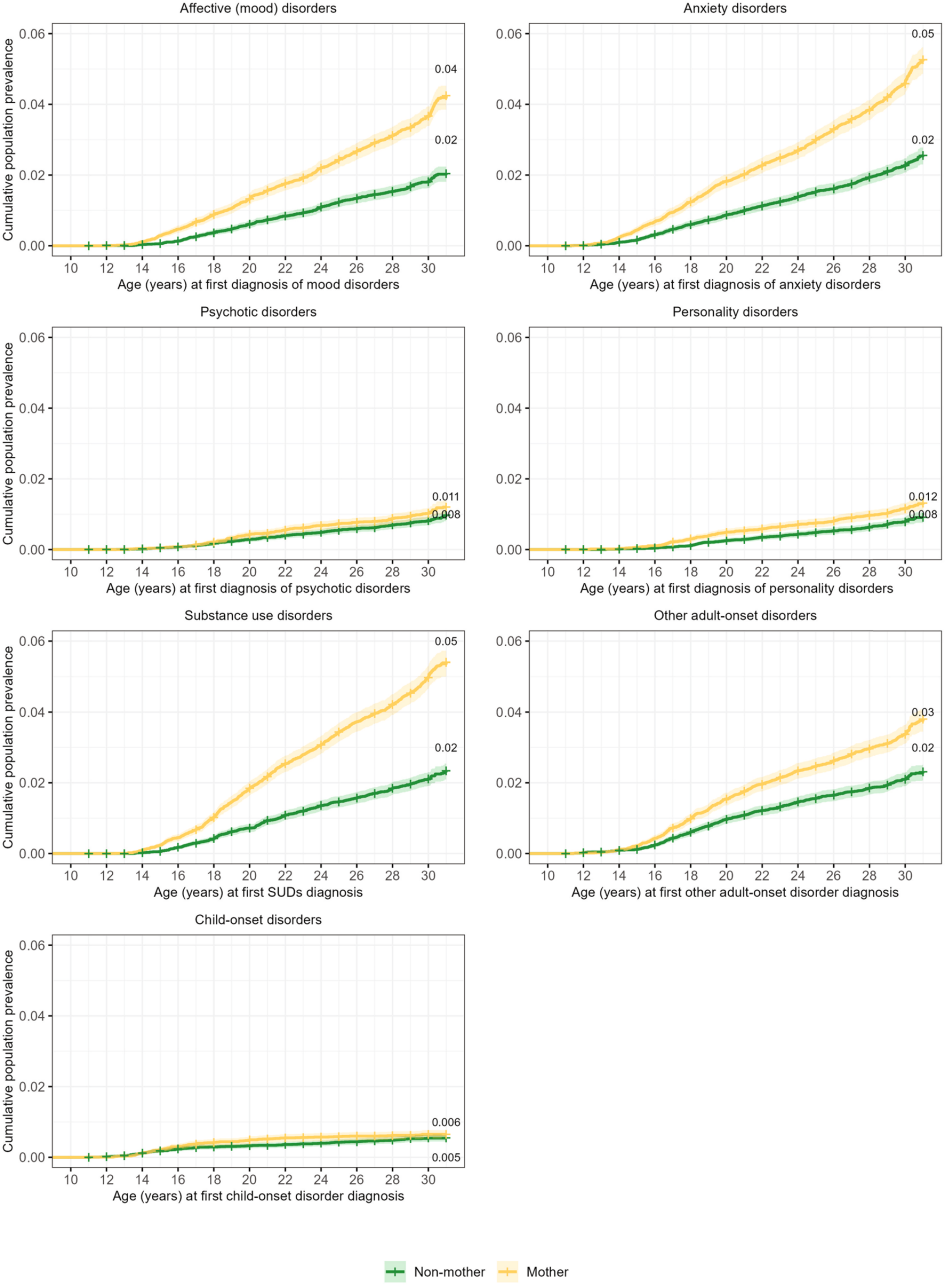
Cumulative prevalence of broad categories of mental disorders up to age 29–31 years by motherhood status

Figure 1 illustrates the cumulative incidence by age for females receiving their first mental disorder diagnosis, while Fig. 2 presents the cumulative incidence curves for each of the broad categories of lifetime mental disorders, stratified by motherhood status. Gray’s test confirms the statistically significant differences in cumulative incidence rates of having at least one mental disorder from age 10–12 to 29–31 years between mothers and non-mothers (*χ*^2^ (1, 40,294) = 460.00, *p* < 0.001). This was also true for all broad diagnostic categories, except for child-onset disorders (*p* < 0.001).

Compared to non-mothers, mental disorders – particularly SUDs, affective (mood), and anxiety disorders – occurred earlier and amplified at a faster rate among mothers. Though cumulative proportions of psychotic disorders and personality disorders differed significantly between mothers and non-mothers, they remained low across age (Fig. 2).

### Comorbidity of mental disorders

**Fig. 3:**
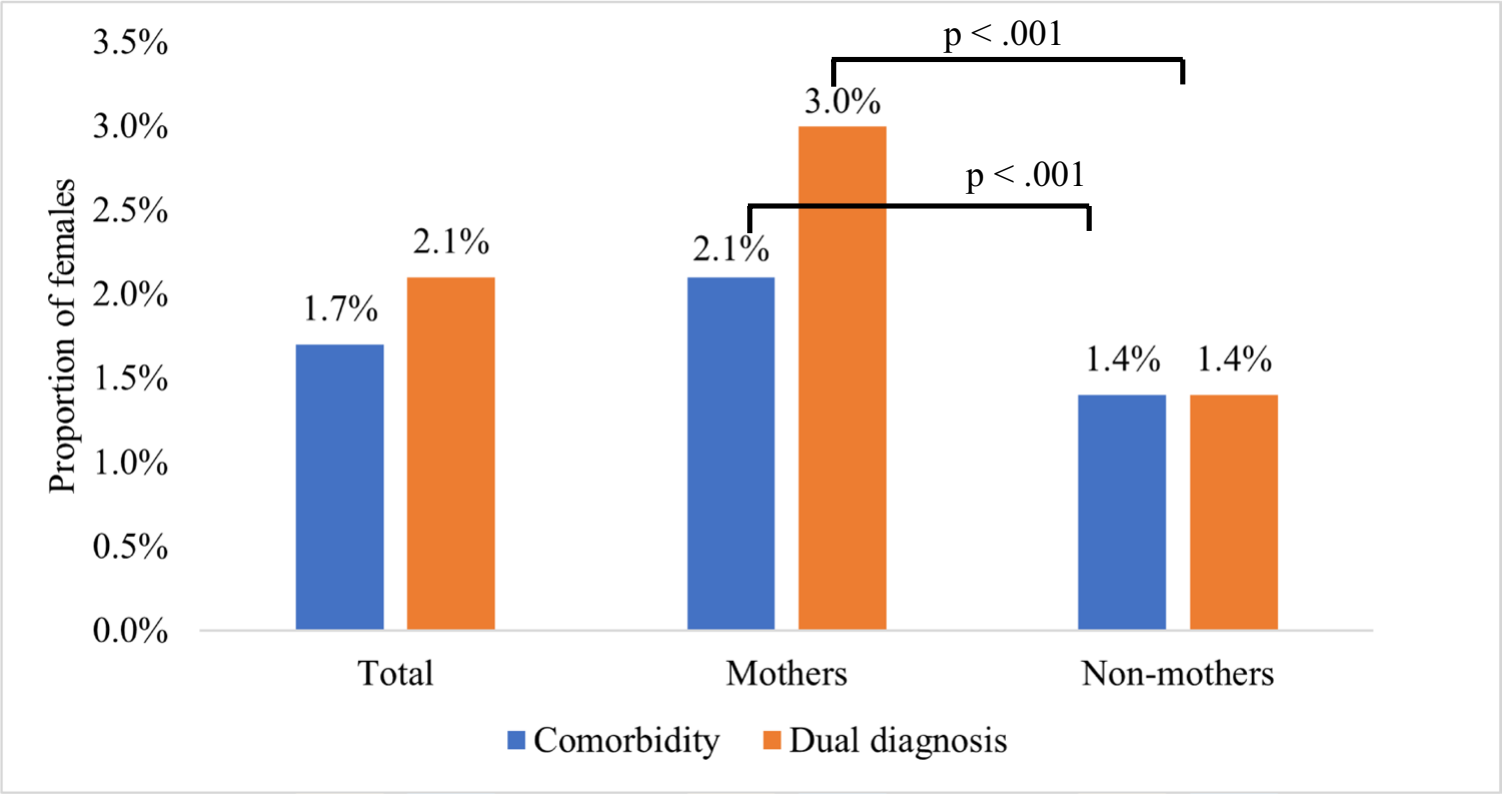
Proportion of females according to their number of mental disorder diagnoses

Figure 3 presents the proportions of all females affected by one or more broad categories of disorders. Among all females, 702 (1.7%) had comorbid diagnoses while 2.1% (*n* = 846) had a dual diagnosis. Compared to non-mothers, mothers had higher rates of comorbid diagnoses (1.4 vs 2.1%; *p* < 0.001, *ϕ*_c_ = 0.06), and a dual diagnosis (1.4 vs 3.0%; *p* < 0.001, *ϕ*_c_ = 0.06).

**Fig. 4:**
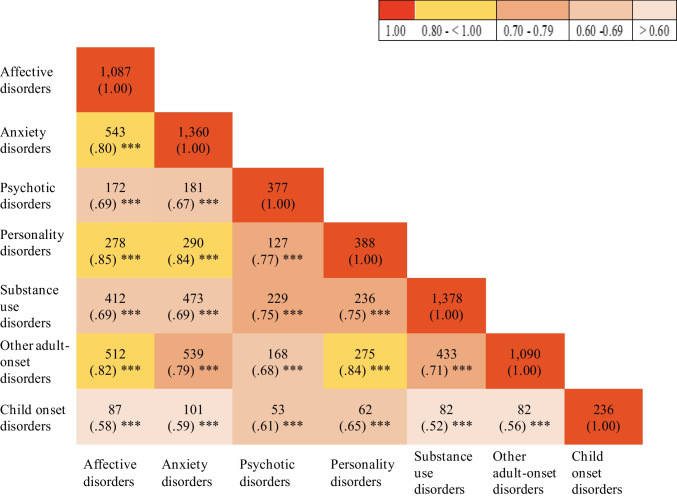
Frequency of comorbidities and tetrachoric correlations between broad categories of mental disorders *Note*. Values in parenthesis indicate correlation coefficients (r_tet_); *p < 0.05, ** *p* < 0.01, *** *p* < 0.001

Tetrachoric correlations between broad categories of mental disorders for all females revealed significant positive correlations across all disorders (*r*_*tet*_ = 0.52– 0.85), with the highest correlations between affective and personality disorders (*r*_*tet*_ = 0.85; Fig. 4). Similar patterns were also observed for tetrachoric correlations separated by motherhood status (see Supplementary Figure S3), indicating a high degree of comorbidity across disorders regardless of motherhood status.

### Sociodemographic and maternal factors associated with mental disorders among mothers

Indigenous mothers and mothers who were never married were more likely to have a mental disorder. Results for maternal factors highlighted two significant variables, age of mother at the first childbirth and number of children. For each increase of one unit of age of mother at the first childbirth, the odds of having mental disorders decreases by 0.88 units and mothers with more children were less likely to have mental disorders than those with one child (AOR: 0.85, 95% CI: 0.76, 0.96) (Table 3).

**Table 3:** Relationship between sociodemographic and maternal factors with prevalence of mental disorder among mothers (*n* = 18,188)

Variables		Mothers with a mental disorder *n* = 1,994	Mothers with no mental disorder *n* = 16,194	Total	*Group diff (X*^2^/U)^a^	β (SE)^b^	AOR (95% CI) ^b^	^b^ Wald *X*^2^
Indigenous status [*n* (%)]	Indigenous	432 (21.7)	1,330 (8.2)	1,762 (9.7)	367.20***	0.55 (0.07)	1.73 (1.52, 1.98)	65.52***
	Non-Indigenous (Ref.)	1,562 (78.3)	14,864 (91.8)	16,426 (90.3)				
Marital status [n (%)]	Never married	1,193 (59.8)	6,046 (37.3)	7,239 (39.8)	374.95***	0.53 (0.05)	1.71 (1.54, 1.89)	99.95***
	Ever married (Ref.)	801 (40.2)	10,148 (62.7)	10,949 (60.2)				
IRSAD^c^	Disadvantaged (1–5)	1,319 (66.6)	9,987 (61.8)	11,306 (62.3)	17.16***	-0.10 (0.05)	0.90 (0.81, 1.00)	3.72
	Advantaged (6–10; Ref.)	662 (33.4)	6,172 (38.2)	6,834 (37.7)				
Age at first birth [mean (S.D.)]		22.20 (3.96)	24.55 (3.92)	24.29 (4.00)	-24.09***	-0.13(0.01)	0.88 (0.87, 0.89)	299.02***
No. of children [n (%)]	Two or more	1,354 (67.9)	9,938 (61.4)	11,292 (62.1)	32.21***	-0.16 (0.06)	0.85 (0.76, 0.95)	7.76**
	One (Ref.)	640 (32.1)	6,256 (38.6)	6,896 (37.9)				

^a^Categorical variables were compared using Pearson *X*^2^ test while continuous variables were compared using Mann–Whitney U Test because of their non-normal distribution

^b^Values derived from a logistic regression model which included 18,140 individuals who have data available for all covariates

^c^Excluding n = 48 cases with missing postcodes

β = unstandardised coefficient; S.E. = standard error of unstandardised coefficient; CI = confidence interval; AOR = adjusted odds ratio, adjusting for all other variables included in the model

Model was significant (*X*^2^ = 840.56, *p* < 0.001), Nagelkerke *R*^*2*^ = 0.09, Hosmer and Lemeshow test was not significant (*p* = 0.06), and the model correctly predicts 89.1% of cases

*p < 0.05, ** *p* < 0.01, *** *p* < 0.001

Of mothers with a mental disorder, 46.9% (*n* = 936) received a mental disorder diagnosis before their first childbirth (Table 4). The average duration between having a first child and receiving a first mental disorder diagnosis was about five years.

**Table 4:** Timing of first inpatient mental disorder diagnosis and childbirth (*n* = 1,994)

	Total [n (%)]	Age at birth of the first child [mean (S.D.)]	Age at the first diagnosis [mean (S.D.)]	Correlation coefficient (95% CI)
Childbirth first	781 (39.2)	20.25 (3.02)	25.47 (3.42)	0.48 (0.42, 0.53) ***
Mental disorder diagnosis first	936 (46.9)	23.58 (3.95)	18.25 (3.56)	0.50 (0.45, 0.54) ***
At the same age	277 (13.9)	23.01 (4.07)	23.02 (4.08)	1.00***

## Discussion

This study found a significant proportion of females experiencing mental disorders, with higher burden among mothers than non-mothers. Mothers who were never married, Indigenous, younger at first childbirth, had greater socio-economic disadvantage and had one child had increased odds of mental disorders. The notable strength of this study is the use of a large population-based sample which addressed sampling bias; thus, enabling the calculation of reliable estimates of lifetime prevalence of different types of mental disorders up to young adulthood among mothers and non-mothers and exploration of correlates of mental disorders among mothers.

This study advances knowledge in four ways. First, we expanded on prior work by estimating prevalence rates of the full range of mental disorders and associated comorbidities, rather than restricting analyses to a few common disorders. This study confirmed prior reports that females experience significant proportions of several, highly correlated mental health comorbidities (Otten et al. 2021; Konrad et al. 2022; Barr et al. 2022), which was also.

evident in separate analyses for mothers and non-mothers. Such strong correlations between mental disorders suggest that mental disorders may share common risk factors, highlighting a need of an integrated, comprehensive approach to effectively address multiple co-occurring mental disorders among females.

Second, important differences in mental health profiles of mothers and non-mothers with mental illness were identified. The high prevalence of SUDs among mothers supports prior evidence on positive association between maternal parenting stress and hazardous alcohol use (Bilsky et al. 2022). Contrast to this, severe mental disorders (e.g., psychotic disorders and child-onset disorders), were more prevalent among non-mothers compared to mothers. Evidence supports that people with such disorders have difficulties in forming romantic relationships and experience higher rates of relationship breakdown than the general population (McCann et al. 2019; White et al. 2021). Furthermore, as these disorders are often diagnosed when they cross a severity threshold and remission requires extended periods (Heckers 2009), it is possible that females with such diagnoses prefer to remain unmarried and childless, although this remains an empirical question in need of further investigation.

Third, in line with previous studies, we found that mothers who were Indigenous, single, young at first birth, and had one child were more likely to have mental illness (Grundy et al. 2019; Owais et al. 2020; Barsisa et al. 2021). Our findings support the argument that lack of support and additional strains placed on single, young mothers, particularly Indigenous mothers, may have compounded the trauma and disadvantage stemming from colonisation, resulting in elevated risk of mental disorders (Owais et al. 2020; Smallwood et al. 2021). Though further research is needed to understand broader factors contributing to mental disorders among mothers, our findings confirmed that the mothers received mental illness at an earlier age and at a faster rate over the age period, when compared to non-mothers. This highlights the importance of early screening coupled with intensive support and treatment of mental disorders for mothers experiencing disadvantage. More importantly, targeted, culturally relevant supports, preferably delivered by Indigenous-led organisations, should be readily available for Indigenous mothers to address their mental disorders and associated risks.

Lastly, this study used a longitudinal dataset to explore timing of the first diagnosis of mental disorders in relation to the first childbirth. Compared to a study by Mowbray et al. (2005), our data documents slightly lower proportion of mothers having their first child before being diagnosed with a mental disorder (39% vs 45%). This may reflect methodological differences between studies, whereby Mowbray et al. (2005) used mothers’ self-reports of mental health contacts and considered all births, while our study relied on administrative data for the first mental disorder diagnosis and first childbirth. Though most studies support increased vulnerability to mental disorders during the first 12 months of childbirth (Munk-Olsen et al. 2006; Leight et al. 2010), our study, consistent with another study from Australia (Woolhouse et al. 2014), indicates that mothers’ risk to mental disorders persists long after the childbirth, suggesting a need of continual mental health support and care beyond the postpartum period. We did not explore how subsequent births impact mental disorders among mothers, nor did we explore socio-demographic differences between mothers based on whether they received a mental disorder diagnosis before or after the first childbirth, highlighting a need for further research.

The present findings should be interpreted in the context of several study-specific limitations. First, as our measurement of mental disorder diagnoses was left-censored at age 10/11 years and right-censored at age 29–31 years, our prevalence estimates do not include women who develop mental disorders in later life, thus, cannot be generalised to females outside this age group. Second, prevalence estimates were based on hospital-derived mental health diagnoses. Although this increased the validity of diagnosis estimates, our prevalence rates are underestimated since not all individuals experiencing mental health symptoms experience or require a hospital admission. Third, our analysis of sociodemographic and maternal correlates of mental disorders was limited to a few variables. Other sociodemographic factors (e.g., educational status, occupation/income, and social support) and maternal factors (e.g., pregnancy and delivery outcomes and complications during pregnancy) that were found to influence mental health among females in previous research were not available for all individuals in the cohort (Biaggi et al. 2016; Patel et al. 2018; Pearson et al. 2019). This prevented us from examining the impact of broader pregnancy and motherhood-related factors on mental disorders of mothers over time, indicating an important avenue for future research. Lastly, Indigenous women and those living in remote and very remote areas less likely to register their children’s births; thus, their motherhood status may be underestimated (Endo et al. 2014).

## Conclusions

This study found that there was significant mental health burden, including mental comorbidities, among females, with mothers’ overrepresentation across all broad categories of mental disorders. Mothers with a mental disorder were more likely to be unmarried, have greater socio-economic disadvantage, Indigenous, younger at first childbirth and have one child. This study contributes to inform and guide the development of priorities for future research and health policy by providing previously lacking prevalence estimates of mental disorders for mothers and non-mothers utilising a representative population-based sample. Regardless of motherhood status, the early onset of mental disorders among females highlights the need for early screening and mental health supports that should be directed towards addressing disadvantage and improving support. Lastly, given the multiple comorbidities among females, there is a need to develop integrated, culturally appropriate interventions targeting multiple mental disorders.

## Data sharing statement

The data for the study are held in Social Analytics Lab (SAL) at Griffith University. Due to privacy, ethical and legal considerations, the QCRC data cannot be shared without direct approval from relevant data custodians and QGSO. Any researcher interested in accessing the data can submit an application to the SAL management committee (socialanalysticslab@griffith.edu.au) with the relevant support and approvals.

### Supplementary information

Below is the link to the electronic supplementary material.

Supplementary file1 (DOCX 44 KB)
