# Supplementary material for: Prevalence of mental disorders among Australian females: Comparison according to motherhood status using Australian birth cohort data
Source: Arch Womens Ment Health. 2024 Feb 20;27(4):625–35. doi: 10.1007/s00737-024-01444-2 (PMC11230990; doi:10.1007/s00737-024-01444-2)
Supplement: Supplementary file 1 — Supplementary file1 (DOCX 44 KB) [file 737_2024_1444_MOESM1_ESM.docx]

**Title: Prevalence of mental disorders among Australian females: Comparison according to motherhood status using Australian birth cohort data**

**Journal name:** Archives of Women’s Mental Health

Diksha Sapkota^1^*, James Ogilvie^1,2^, Susan Dennison^1,2^, Carleen Thompson^1,2^, Troy Allard^1,2^

^1^Griffith Criminology Institute, Griffith University, Australia

^2^School of Criminology and Criminal Justice, Griffith University, Australia

*Corresponding author

Diksha Sapkota, Griffith Criminology Institute, Griffith University, 176 Messines Ridge Road, Mount Gravatt, QLD 4122, Australia.

Email: [d.sapkota@griffith.edu.au](mailto:d.sapkota@griffith.edu.au)

**Supplementary Table S1 Mental disorder diagnostic groups and their subdivisions classifications by ICD-10-AM codes**

| **Broad diagnostic group** | **Detailed diagnostic subdivisions** | **ICD-10 codes** |
| --- | --- | --- |
| Mood (affective) disorders | Depression (includes recurrent depressive disorder, cyclothymia, dysthymia, excludes disorders with psychotic symptoms) | F32.0 – F32.9 (excluding F32.3); F33.0 – F33.9 (excluding F33.3); F34.0; F34.1 |
|  | Bipolar (excludes disorders with psychotic symptoms as they are kept under psychotic affective disorders) | F31 (excluding F31.2, F31.5) |
|  | Other affective disorders | F30.0- F30.9 (excluding F30.2); F34.8; F34.9; F38; F39 |
| Anxiety disorders | Phobic anxiety disorders | F40.0 – F40.9 |
|  | Obsessive compulsive disorders | F42.0 – F42.9 |
|  | Reaction to severe stress | F43.0; F43.1; F43.8; F43.9 |
|  | Adjustment disorders | F43.2 |
|  | Other anxiety disorders (e.g., panic disorders, dissociative disorders, somatoform disorders, other neurotic disorders) | F41.0- F41.9; F44.0- F44.9; F45.0-F45.9; F48.0-F48.9 |
| Substance use disorders | Mental and behavioural disorders due to use of alcohol | F10 |
|  | Mental and behavioural disorders due to use of substances | F11; F12; F13; F14; F15; F16; F17; F18; F19 |
| Psychotic disorders | Schizophrenia including schizoaffective and other psychotic disorders | F20; F21; F22; F23; F24; F25; F26; F27; F28; f29 |
|  | Psychotic affective disorders | F30.2; F31.2; F31.5; F32.3; F33.3 |
|  | Psychotic disorders related to substance use | F10.5; FF11.5; F12.5; F13.5; F14.5; F15.5; F16.5; F17.5; F18.5; F19.5; F19.7 |
| Other adolescent and/or adult-onset disorders | Eating disorders | F50 |
|  | Other adolescent and/or adult-onset disorders disorders (e.g., sleep disorders, sexual dysfunction, psychological or behavioural disorders, abuse of non-dependence producing substances; habit and impulse disorders; gender identity disorders; sexual disorders; other unspecified disorders) | F51; F52; F54; F55; F59; F63, F64, F65, F66; F99 |
|  | Mental and behavioral disorders associated with puerperium not classified elsewhere | F53 |
|  | Self-harm and suicidal ideation | R45.8, X60-X84 |
|  | Organic disorders (e.g., dementia, disorders due to brain damage or dysfunction, amnesic syndrome) | F00; F01; F02; F03; F04; F05; F06; F07; F09 |
| Child-onset disorders | Mental retardation | F70; F71; F72; F73; F78; F79 |
|  | Disorders of psychological development (e.g., disorders of speech and language; pervasive developmental disorders) | F80; F81; F82; F83; F84; F88; F89 |
|  | Child emotional and behavioural disorders (e.g., conduct and hyperkinetic disorders, mixed disorders of conduct and emotion, emotional disorders, disorders of social functioning, tic disorders) | F90; F91; F92; F93; F94; F95; F98 |
| Personality disorders | Disorders of adult personality and behaviour (e.g., specific, mixed, and other personality disorders, enduring personality change, other disorders of adult personality and behaviour, unspecified disorder of adult personality and behaviour) | F60, F61, F62, F68, F69 |

**Supplementary Table S2 Sociodemographic and mental-health profile differences of females in 1983/84 birth cohorts by motherhood status (n = 3,133)**

| **Variables** | | **Mothers** | **Non mothers** | **Total** | ***Group diff (X*^2^/*U*)** ^a^ | **β (SE)**^b^ | **AOR (95% CI)** ^b^ | ^b^ **Wald *X*^2^** |
| --- | --- | --- | --- | --- | --- | --- | --- | --- |
| Indigenous status [n (%)] | Indigenous | 432 (21.7) | 138 (12.1) | 570 (18.2) | 44.41*** | 0.77 (0.12) | 2.17 (1.72, 2.73) | 43.20*** |
|  | Non-Indigenous | 1,562 (78.3) | 1,001 (87.9) | 2,563 (81.8) |  |  |  |  |
| Marital status [n (%)] | Ever married | 801 (40.2) | 143 (12.6) | 944 (30.1) | 262.60*** | 1.72 (0.11) | 5.57 (4.52, 6.86) | 259.46*** |
|  | Never married | 1,193 (59.8) | 996 (87.4) | 2,189 (69.9) |  |  |  |  |
| Remoteness of residence [n (%)]^c^ | Regional/ Remote areas | 1079 (54.4) | 521 (46.1) | 1,600 (51.4) | 20.09*** | 0.05 (0.09) | 1.06 (0.88, 1.26) | 0.35 |
|  | Major cities | 904 (45.6) | 610 (53.9) | 1,514 (48.6) |  |  |  |  |
| Index of relative disadvantage for residence [mean (S.D.)]^d^ | | 4.79 (4.93) | 5.57 (3.91) | 5.88 (3.14) | -8.37*** | -0.14 (0.02) | 0.87 (0.84, 0.90) | 64.91*** |
| Anxiety disorders [n (%)] | | 853 (42.8) | 507 (44.5) | 1,360 (43.4) | 0.89 | 0.01 (0.09) | 1.01 (0.84, 1.22) | 0.01 |
| Affective (mood disorders) [n (%)] | | 683 (34.3) | 404 (35.5) | 1,087 (34.7) | 0.47 | 0.05 (0.10) | 1.05 (0.86, 1.28) | 0.27 |
| Psychotic disorders [n (%)] | | 192 (9.6) | 185 (16.2) | 377 (12.0) | 29.95*** | -0.55 (0.13) | 0.58 (0.45, 0.75) | 17.50** |
| Personality disorders [n (%)] | | 213 (10.7) | 175 (15.4) | 388 (12.4) | 14.65*** | -0.09 (0.14) | 0.92 (0.70, 1.21) | 0.39 |
| Substance use disorders [n (%)] | | 909 (45.6) | 469 (41.2) | 1,378 (44.0) | 5.72* | 0.09 (0.14) | 1.10 (0.84, 1.43) | 0.44 |
| Child-onset disorders [n (%)] | | 117 (5.9) | 119 (10.5) | 236 (7.5) | 21.85*** | -0.52 (0.16) | 0.59 (0.44, 0.81) | 10.80** |
| Other adult-onset disorders [n (%)] | | 625 (31.3) | 465 (40.8) | 1,090 (34.8) | 28.72*** | -0.35 (0.17) | 0.71 (0.58, 0.86) | 11.93*** |
| Dual diagnosis [n (%)] | | 546 (27.4) | 300 (26.3) | 846 (27.0) | 0.4 | 0.35 (0.17) | 1.41 (1.01, 1.97) | 4.17* |
| Co-morbid diagnosis [n (%)] | | 385 (19.3) | 317 (27.8) | 702 (22.4) | 30.29*** | -0.13 (0.13) | 0.88 (0.68, 1.13) | 1.03 |
| Age at first mental health related hospitalisation [mean (S.D.)] | | 21.74 (4.93) | 21.37 (4.92) | 21.61 (4.93) | 1.95 | 0.004 (0.01) | 1.00 (0.99, 1.02) | 0.20 |

^a^ Categorical variables were compared using Pearson *X*^2^ test while continuous variables were compared using Mann-Whitney U test (as they were non-normally distributed)

^b^ Values derived from a logistic regression model which included 3114 individuals who have data available for all covariates

^c^ Excluding n = 19 cases with unknown residence

^d^ Excluding n = 18 cases with unknown SEIFA index

β = unstandardised coefficient; S.E. = standard error of unstandardised coefficient; CI = confidence interval; AOR = adjusted odds ratio, adjusting for all other variables included in the model

Model was significant (*X*^2^ = 531.01, p < .001), Nagelkerke *R^2^* = 0.22, Hosmer and Lemeshow test was not significant (*p* = .733), and the model correctly predicts 69.6% of cases

**p* < .05, ** *p* < .01, *** *p* < .001

| Affective disorders | 683  (1.00) |  |  |  |  |  |  |  | Affective disorders | 404  (1.00) |  |  |  |  |  |  |
| --- | --- | --- | --- | --- | --- | --- | --- | --- | --- | --- | --- | --- | --- | --- | --- | --- |
| Anxiety disorders | 324  (.75***) | 853  (1.00) |  |  |  |  |  |  | Anxiety disorders | 219  (.84***) | 507  (1.00) |  |  |  |  |  |
| Psychotic disorders | 84  (.63***) | 94  (.63***) | 192  (1.00) |  |  |  |  |  | Psychotic disorders | 88  (.75***) | 87  (.71***) | 185  (1.00) |  |  |  |  |
| Personality disorders | 153  (.82***) | 163  (.82***) | 64  (.74***) | 213  (1.00) |  |  |  |  | Personality disorders | 125  (.88***) | 127  (.86***) | 63  (.79***) | 175  (1.00) |  |  |  |
| Substance use disorders | 258  (.64***) | 300  (.64***) | 128  (.74***) | 131  (.71***) | 909  (1.00) |  |  |  | Substance use disorders | 154  (.74***) | 173  (.73***) | 101 (.78***) | 105 (.80***) | 469  (1.00) |  |  |
| Other adult-onset disorders | 287  (.78***) | 306  (.75***) | 81  (.63***) | 141  (.80***) | 260  (.57***) | 625  (1.00) |  |  | Other adult-onset disorders | 225  (.87***) | 233  (.84***) | 87  (.73***) | 134 (.88***) | 173 (.75***) | 465  (1.00) |  |
| Child onset disorders | 52  (.59***) | 45  (.56***) | 23  (.56***) | 32  (.64***) | 49  (.52***) | 46  (.57***) | 117  (1.00) |  | Child onset disorders | 35  (.57***) | 48  (.63***) | 30  (.65***) | 30  (.66***) | 33  (.53***) | 36  (.56***) | 119  (1.00) |
|  | Affective disorders | Anxiety disorders | Psychotic disorders | Personality disorders | Substance use disorders | Other adult-onset disorders | Child onset disorders |  |  | Affective disorders | Anxiety disorders | Psychotic disorders | Personality disorders | Substance use disorders | Other adult-onset disorders | Child onset disorders |


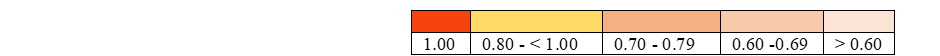


**Fig. S3** Frequency of comorbidities and tetrachoric correlations between different types of mental disorders, by motherhood status

Note. Values in parenthesis indicate correlation coefficients; *p < .05, ** p < .01, *** p < .001
